# Conjugation in *Euplotes raikovi* (Protista, Ciliophora): New Insights into Nuclear Events and Macronuclear Development from Micronucleate and Amicronucleate Cells

**DOI:** 10.3390/microorganisms8020162

**Published:** 2020-01-23

**Authors:** Ruitao Gong, Yaohan Jiang, Adriana Vallesi, Yunyi Gao, Feng Gao

**Affiliations:** 1 Institute of Evolution & Marine Biodiversity, Ocean University of China, Qingdao 266003, China; gongruitao0306@163.com (R.G.); jiangyaohan2020@163.com (Y.J.); yunyi_gao@163.com (Y.G.); 2 Key Laboratory of Mariculture (Ocean University of China), Ministry of Education, Qingdao 266003, China; 3 Laboratory of Eukaryotic Microbiology and Animal Biology, University of Camerino, 62032 Camerino, Italy; adriana.vallesi@unicam.it

**Keywords:** amicronucleate cells, ciliate, conjugation, *Euplotes raikovi*, life cycle

## Abstract

Ciliates form a distinct group of single-celled eukaryotes that host two types of nuclei (micro and macronucleus) in the same cytoplasm and have a special sexual process known as conjugation, which involves mitosis, meiosis, fertilization, nuclear differentiation, and development. Due to their high species diversity, ciliates have evolved different patterns of nuclear events during conjugation. In the present study, we investigate these events in detail in the marine species *Euplotes raikovi*. Our results indicate that: (i) conjugation lasts for about 50 h, the longest stage being the development of the new macronucleus (ca. 36 h); (ii) there are three prezygotic micronuclear divisions (mitosis and meiosis I and II) and two postzygotic synkaryon divisions; and (iii) a fragment of the parental macronucleus fuses with the new developing macronucleus. In addition, we describe for the first time conjugation in amicronucleate *E. raikovi* cells. When two amicronucleate cells mate, they separate after about 4 h without evident nuclear changes; when one amicronucleate cell mates with a micronucleate cell, the micronucleus undergoes regular prezygotic divisions to form migratory and stationary pronuclei, but the two pronuclei fuse in the same cell. In the amicronucleate cell, the parental macronucleus breaks into fragments, which are then recovered to form a new functional macronucleus. These results add new information on the process of conjugation in both micronucleate and amicronucleate *Euplotes* cells.

## 1. Introduction

Ciliates are single-celled eukaryotic microorganisms that can be found in diverse habitats across the globe [1,2,3,4,5,6]. They are unique among eukaryotes for two distinctive features: the presence of two types of nuclei and the sexual process typically represented by conjugation. In each cell, a germline micronucleus (MIC), which is inactive during the vegetative life coexists with a somatic polyploid macronucleus (MAC), which controls the general functions of the cell [7]. The genome of the MIC is arranged into typical eukaryotic chromosomes, while the genome of the MAC is composed by centromere-less chromosomes amplified in hundreds of copies [7,8,9,10,11]. Because of these characters, ciliates are used as model organisms in a wide range of disciplines, including cytology, evolutionary biology, and genetics [12,13,14,15,16,17].

Conjugation has been widely studied since it was discovered in *Paramecium* [18] and has been described in many ciliates, such as *Tetrahymena*, *Chilodonella*, *Oxytricha*, and *Euplotes* [19,20,21,22,23,24]. During the sexual process for most species, two cells unite in a mating pair, fuse their cell membranes to form a cytoplasmic bridge, and exchange gametic pronuclei derived from MIC meiotic divisions before separating. In each mating cell, gametic nuclei merge to form the synkaryon; meanwhile, the parental MAC is destroyed and exconjugants have to replace their ‘old’ nuclear apparatus with the mitotic products of the synkaryon [19,22,23,24,25,26,27,28]. A new MAC genome is then generated through complex mechanisms of chromosome fragmentation, DNA elimination, and amplification [9,10,29,30]. For these reasons, the functional advantage of sex in ciliates is related to nuclear reorganization, which allows cells to start a new life cycle, whereas reproduction is carried out simply by binary fission.

Conjugation is genetically controlled by the so-called ‘mating type systems’ [31]. In general, two cells having non-identical mating types can undergo successful conjugation, although some species are capable of mating with individuals of the same mating type (homotypic pairing). The number of mating types varies greatly among species, from two as in *Paramecium aurelia* [18], to seven as in *Tetrahymena thermophila* [32], to many as in *Euplotes* [33,34,35,36]. In *Euplotes*, each mating type is determined by the allele combination at the ‘mating type locus’ of the germline MIC [37,38,39]. These *mat* alleles control the synthesis of waterborne signaling molecules known as pheromones, which are able to promote both sex and proliferation according to the type of interaction with specific cell surface receptors [37].

Conjugation in *E. raikovi* was first described in 1981 [40]. In this study, we reanalyze the entire process in more detail, and report for the first time mating in amicronucleate cells that, surprisingly, are able to form pairs with both micronucleate (mic) and amicronucleate (amic) cells.

## 2. Materials and Methods

### 2.1. Cell Culturing and Species Identification

Five *Euplotes raikovi* strains were used in this study. The wild-type strains U and V were collected in July 2015 from the Silver Sand Beach of Qingdao (35°55′ N, 120°12′ E), China (water temperature 24 °C, salinity ~30‰); strain F1 was obtained as sexual offspring of mating pairs between U and V strains; type-I and type-XIII cells were selected on the basis of their homozygous *mat*-allelic combination, as indicated by the presence of a single type of MAC pheromone gene and by secretion of a single type of pheromone (E*r*-1 and E*r*-13, respectively) [41] (and unpublished data). Clonal cultures were maintained at 22 °C in sterilized seawater, using *Escherichia coli* as food source. Species identification was based on morphological characters observed both *in vivo* and after protargol staining [42], and on small subunit ribosomal (SSU) rRNA gene sequence determination according to Wang et al. 2019 [12].

### 2.2. Mating Pair Induction and Analysis of Nuclear Events in Mating Cells

Conjugation was induced by mixing cells that were previously centrifuged at 500× *g* for 3 min, suspended in fresh seawater at a concentration of 4000 cell/mL, and starved for 36–40 h. Mating pairs were picked out just after their formation and suspended in fresh seawater (time 0). Samples were then collected every 30 min, incubated with 50% formalin solution (1:1 *v*/*v*) at room temperature for 1 min, and stained with Hoechst 33342 at 1.25 µg/mL final concentration (Beyotime Institute of Biotechnology, Haimen, Jiangsu, China) to visualize the nuclei. Cells were then transferred to a glass microscope slide, covered with a coverslip, and observed under a “ZEISS AXIO Imager. D2” fluorescence microscope, equipped with an Axiocam 506 camera for photographic documentation. For each time point, 30–50 pairs were recorded.

### 2.3. Phylogenetic Analyses

The SSU-rRNA gene sequence of strain U was aligned with other 52 *Euplotes* sequences deposited in the NCBI database, using the MUSCLE algorithm (https://www.ebi.ac.uk/Tools/msa/muscle/). The SSU-rRNA genes of *Diophrys scutum* (MQ603644) and *Aspidisca aculeata* (EF123704) were selected as outgroups. The alignment was manually modified using BioEdit v7.2.3 [43] to generate a matrix of 55 taxa with 1670 nucleotide sites. Maximum likelihood (ML) and Bayesian inference (BI) analyses were performed in CIPRES Science Gateway (http://www.phylo.org/sub_sections/portal/) [44]. The ML tree was constructed using RAxML-HPC2 on XSEDE v.8.2.12 with GTR+I+G model and 1000 bootstrap replicates [45]. BI analysis was performed using MrBayes on XSEDE v.3.2.6 with GTR+I+G model, which was selected by MrModeltest v2.2 [46] and PAUP v4.0b10 [47]. Markov chain Monte Carlo (MCMC) simulations were run for 10,000,000 generations with a frequency of 100 generations and a burn-in of 10,000 trees. A majority rule consensus tree with posterior probabilities (PP) was constructed by all remaining trees. Tree topologies were visualized with MEGA v10.0.5 [48].

## 3. Results

### 3.1. Mating Interactions

Conjugation was induced by mixing cells of different strains in all pairwise combinations (Table 1). After the so-called ‘waiting period’ of about 2 h, stable mating pairs were regularly observed by mixing cells of strain U with all the other strains, while no pairs were observed mixing strains V, F1, and type-XIII, implying that they belong to the same mating type. Therefore, U × XIII, U × F1, and I × XIII cell mixtures were used to analyze nuclear events during conjugation described in the following sections.

### 3.2. Prezygotic Divisions to Synkaryon Formation in Mating Cells

**Figure 1:**
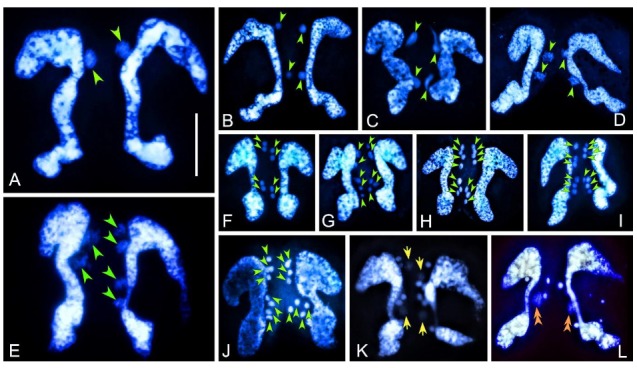
Nuclear events in conjugating pairs of *Euplotes raikovi* after Hoechst 33342 staining. (**A**) At the beginning of conjugation, the micronucleus (MIC) of each mating cell swells and migrates out of the concavity of the macronucleus (MAC). (**B**) After the first prezygotic division of the MIC. (**C**–**F**) Various stages of the first meiosis division. Cells in (**C**) is the zygotene stage of the first meiosis: the chromatin polymerizes into a typical ‘bouquet’ shape. In (**E**), cells have different numbers of nuclei due to asynchronous division. (**G**–**J**) Mating cells during the second meiotic division with different numbers of nuclear products due to asynchronous division. In (**J**), end of the second meiotic division results in eight haploid nuclei. (**K**) In each cell, two pronuclei (indicated by yellow arrows) swell while the other nuclei degenerate. (**L**) Synkaryon formation after exchange and fusion of pronuclei (orange double-arrowheads). Green arrowheads indicate nuclei derived from prezygotic divisions; yellow arrows indicate the pronuclei; orange double-arrowheads indicate the synkaryon. Scale bar = 20 μm.

Following cell pairing, the MIC in each mating partner starts to swell, migrates out of the concavity of the “C”-shaped MAC, and divides by mitosis (first prezygotic division), which lasts about 3 h (Figure 1A,B). Then, the two mitotic products undergo a classical two-step meiosis (second and third prezygotic divisions), which takes about 4 h (Figure 1C–J). It was observed that meiotic divisions can occasionally (ca. 20%–30%) be asynchronous: of the two mating cells, one may have finished the first meiotic division and contains four haploid nuclei, while the other has yet to start and still contains the two mitotic products (Figure 1E). Furthermore, the four haploid nuclei in the same cell do not divide simultaneously during the second meiotic division (Figure 1G–I).

At the end of meiosis, each mating cell contains eight haploid nuclei: four in the anterior part and four in the posterior part of the cell (Figure 1J). For each anterior and posterior group of nuclei, one out of four swells, while the other three degenerate (Figure 1K). The two swollen nuclei represent the migratory and stationary pronuclei. After the reciprocal exchange of the migratory pronucleus, its fusion with the stationary one generates the synkaryon in each mating cell (Figure 1L). The degenerating meiotic products that have accumulated on the edge of the cell are still visible at this stage. This process takes about 1 h.

### 3.3. Postzygotic Divisions and Development of the New Nuclear Apparatus in Exconjugant Cells

**Figure 2:**
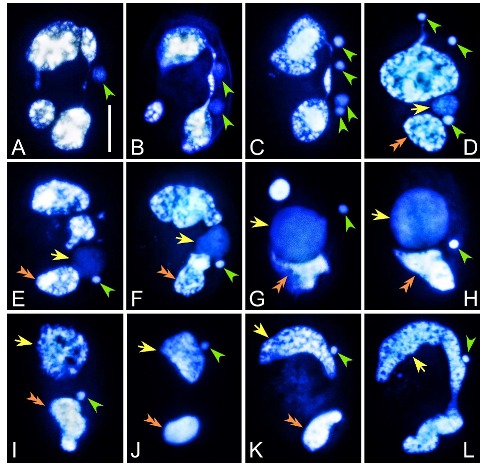
Nuclear events in the exconjugants of *Euplotes raikovi* after Hoechst 33342 staining. (**A**) After mating pair separation, the parental MAC breaks into fragments. (**B**,**C**) First and second postzygotic divisions of the synkaryon. (**D**–**H**) One out of the four products (usually the third one from anterior to posterior) of the synkaryon divisions swells and differentiates into the MAC anlage. The other three products degenerate. The anterior part of the parental MAC gradually degrades. (**I**–**L**) The MAC anlage gradually reorganizes, fuses with the remnant parental MAC, and finally develops into the new MAC (**L**). Green arrowheads: division products of the synkaryon and new MIC. Yellow arrows: MAC anlage and new MAC. Orange double-arrowheads: fragment of the parental MAC which fuses with the developing new MAC. Scale bar = 20 μm.

Mating cells usually separate within 1 h after synkaryon formation. During this time, the residual meiotic products completely degenerate, and the parental MAC starts degrading into a large anterior and a small posterior fragment (Figure 2A). In the 1.5 h following mating pair separation, the synkaryon of each exconjugant cell divides twice by mitosis (first and second postzygotic divisions), forming four nuclei, two of which localize in the anterior part and two in the posterior part of the cell (Figure 2B,C). The two anterior postzygotic nuclei degenerate; of the two posterior postzygotic nuclei, one becomes the new MIC and the other differentiates into the new developing MAC, called anlage (Figure 2D), which is distinguishable from the parental MAC by being weakly stained. The MAC anlage increases in size and gradually moves from the middle to the anterior part of the cell (Figure 2E–H). Meanwhile, the anterior fragment of the parental MAC progressively disappears, while the small posterior part remains and fuses with the new developing MAC (Figure 2E–H, Figure S1A–D). About 36 h after anlage formation, the new MAC differentiates into the typical ‘C’ shape (Figure 2I–L). The nuclear division occurs, as usual, during the first postconjugational cell division. We did not observe the degeneration of the fused parental MAC by the completion of the first postconjugational division (Figure S1E–I).

### 3.4. Conjugation in Amicronucleated Cells

**Figure 3:**
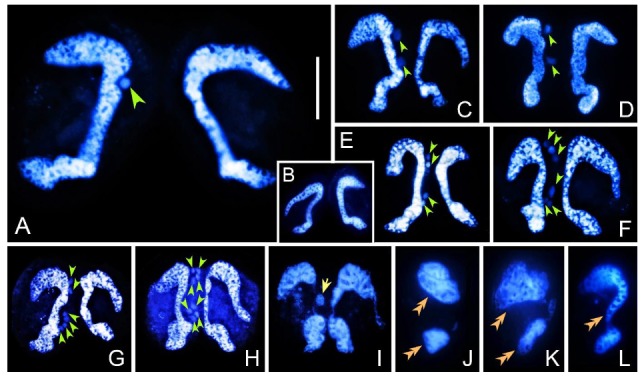
Nuclear events during conjugation of *Euplotes raikovi* amic cells pairing with amic or mic cells. (**A**) Mating pair between an amic and a mic cell. (**B**) Mating pair formed by two amic cells. (**C**) First prezygotic division in the mic cell. (**D**–**H**) Mating cells in the first and second meiotic division, resulting in eight nuclear products in the mic cell (**H**). (**I**) Synkaryon formation in the mic cell. Parental MAC starts to fragment in both cells. (**J**–**L**) Regeneration of MAC in the amic cell after separation. Scale bar = 20 μm.

Cells without a micronucleus were found in *E. raikovi* strains U, F1, and XIII. Amicronucleate (amic) cells have the same growth rate and viability as the micronucleate (mic) cells and can mate with both amic and mic cells (Figure 3). When two amic cells mate, they remain united for about 3–4 h. During this period their MACs do not change visibly (Figure 3B), and after separation the two exconjugant amic cells divide normally.

When an amic cell mates with a mic cell, the latter completes the prezygotic divisions leading to the production of migratory and stationary pronuclei (Figure 3A,C–G). However, we did not observe the exchange of the migratory pronucleus with the amic partner. It is highly possible that migratory pronucleus remains in the mic cell and fuses with the stationary one to form the synkaryon (Figure 3I). Then, the two cells separate and the nuclear apparatus in the mic cell develops as usual. It is noteworthy that there is no synkaryon in the amic cell and its MAC does not fragment as severely as the normal one but typically breaks into two parts (Figure 3J). Both parts regenerate and are reshaped into a typical “C” shape within about 50 h after conjugation (Figure 3K–L).

## 4. Discussion

### 4.1. Nuclear Events during Conjugation in Micronucleate E. raikovi Cells: Comparison with Other Euplotes Species

**Figure 4:**
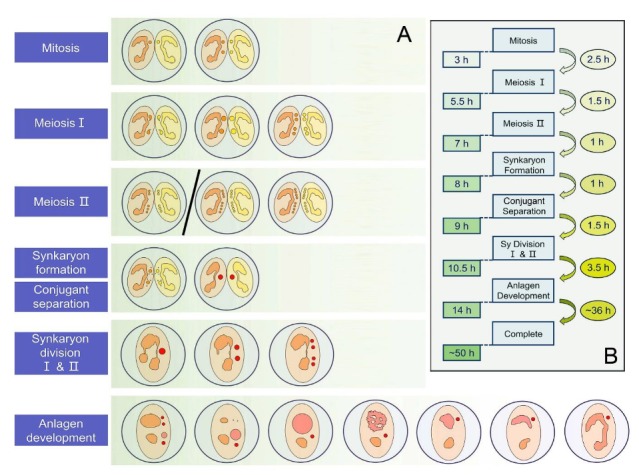
Summary of the nuclear events occurring during conjugation in *Euplotes raikovi*. (**A**) Schematic diagrams showing the nuclear events at each step of conjugation. (**B**) Time chart of each step of conjugation. The initial formation of mating pairs was taken as time 0 of the process. The number on the left indicates the time point from initial pair formation. The number on the right indicates the time taken by each step.

**Figure 5:**
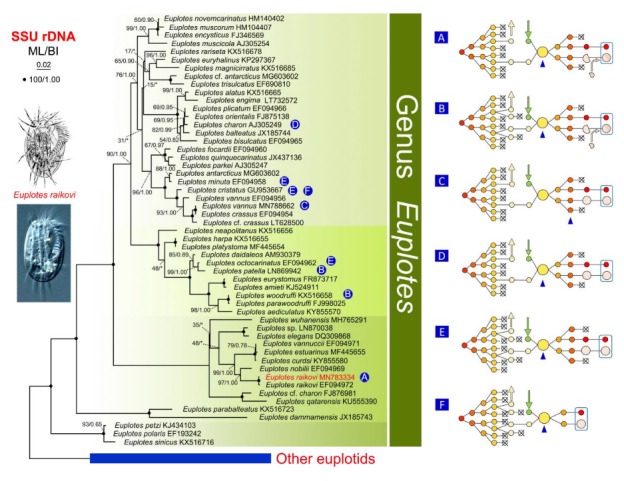
Maximum likelihood (ML) tree of the family Euplotidae based on SSU-rRNA gene sequences. *Diophrys scutum* (MQ603644) and *Aspidisca aculeata* (EF123704) were used as outgroup species. Numbers at nodes represent the bootstrap values of maximum likelihood (ML) out of 1000 replicates and posterior probability of Bayesian analysis (BI). Asterisk (*) indicates disagreement between ML and BI analyses. The scale bar corresponds to 5 substitutions per 100 nucleotide positions. (**A**–**F**) Different patterns of the nuclear events during conjugation in *Euplotes* species (reviewed from [23,24,27,28,33,49,50,51]).

In the present study, we investigated the nuclear events during conjugation in *E. raikovi*. The whole process lasts for about 50 h, including three prezygotic MIC divisions (mitosis, meiosis I, and meiosis II) and two postzygotic synkaryon divisions after pair separation (Figure 4). The longest stage is the development of the new MAC (about 36 h). Comparing nuclear events during conjugation in *E. raikovi* with those in other *Euplotes* species reveals differences in (i) patterns of prezygotic and postzygotic nuclear divisions, (ii) time points of conjugants separation, and (iii) behaviors of the parental MAC during the development of the new MAC (Figure 5).

In *E. raikovi* and *E. vannus*, the MIC undergoes three prezygotic divisions for generating gametic pronuclei [24]. In *E. woodruffi*, *E. patella*, *E. octocarinatus*, *E. affinis*, *E. minuta*, *E. cristatus*, and *E. charon*, four prezygotic divisions occur in the MIC, with an additional mitotic division following meiosis [27,28,33,36,49,50,51]. However, *E. raikovi* shows a peculiar feature in that meiotic divisions are not always synchronized. Asynchronous divisions are manifested in cells from the same mating pair, thus implying that each partner has an autonomous clock that functions independently from the other. All the *Euplotes* species studied so far undergo two postzygotic nuclear divisions, except for *E. cristatus*, which generally undergoes one or occasionally two postzygotic divisions, and *E. charon* in which the synkaryon usually undergoes three divisions [23,51].

The conjugants separate at different time points in different *Euplotes* species. *E. vannus* cells remain united until the synkaryon divides twice, while in other *Euplotes* species mating pairs separate immediately after pronuclei exchange and synkaryon formation (Figure 5). The time point of conjugants separation is also diverse in other ciliates as reviewed in Jiang et al., 2019 [24].

The behavior of the parental MAC during the development of the new MAC varies among different species of *Euplotes*. First, the time point of the parental MAC fragmentation differs. The parental MAC in *E. vannus* and *E. cristatus* starts to fragment during the first meiotic division. Conversely, the parental MAC in *E. raikovi* and other *Euplotes* species starts to break into irregular and polymorphic bodies after formation of the zygotic nucleus. More importantly, the contribution of the parental MAC to the development of the new MAC also varies among different species of *Euplotes*. In the exconjugants of *E. vannus*, *E. octocarinatus*, *E. affinis*, *E. minuta*, *E. charon*, and *E. cristatus*, all fragmented parts of the parental MAC degenerate completely [23,24,27,28,33,49,50,51]. Some researchers reported that the small posterior fragment of the parental MAC regenerates and fuses with the developing new MAC in the exconjugants of *E. woodruffi* [27] and *E. patella* [28]. However, some other researchers disagreed with this observation and stated that the fused fragment of the parental MAC degrades again during the first postconjugational division in the two *Euplotes* species [52,53]. It is possible that the previous researchers might neglect some important events. The other possibility is that species identification was in question and they used different species even though the name was the same. In *E. raikovi*, we clearly observe the fusion of the parental MAC within the new MAC (Figure S1A–D). We checked its first postconjugational division and did not observe the demarcation or degradation of the fused old MAC (Figure S1E–I). In any case, the process of conjugation may vary dramatically in different species and we cannot overgeneralize this phenomenon in other ciliates even within *Euplotes* species.

It is still unclear what roles the residual parental MAC plays in the development of the new MAC. It has been tested in *E. aediculatus* that the fragments of the parental MAC are necessary for normal nuclear and cortical development [54]. Studies in *E. woodruffi* have shown that RNA synthesis is always present in the parental MAC fragments until the new MAC develops to T-shape [55]. Moreover, the fragments of the parental MAC can regenerate once the MAC anlage is damaged [56]. In the present study, the fragments of the parental MAC reshape into a typical “C” shape in the amic cell, providing further evidence to their ability to regenerate. More importantly, it has been proved in some ciliates that new MAC is developed under the indirect influence of the old MAC through small RNAs or templated RNAs [11,57]. The fusion of the old MAC into the new MAC in some *Euplotes* species, no matter if it is degraded later or not, provides another possibility that the residual parental DNA may have a direct influence on the new MAC, e.g., acting as template to guide rearrangements (deletion, permutation, and inversion) of corresponding MIC DNA sequences. More evidence and molecular mechanisms behind this phenomenon require further investigation.

In order to investigate the evolutionary relationship of the above phenomena, e.g., patterns of prezygotic nuclear divisions, and the retention of partial parental MAC in the new MAC, we constructed phylogenetic trees based on SSU-rRNA gene sequence of the family Euplotidae (Figure 5). Species sharing the same pattern of conjugation events are dispersed all over the trees. For example, *E. raikovi* and *E. vannus*, which have three prezygotic divisions are clearly separated, as are those species with four nuclear prezygotic divisions; the species that retain partial parental MAC in the new MAC also do not group together. This suggests that these characters evolved independently and do not reflect evolutionary relationships among *Euplotes* species.

### 4.2. Conjugation in Amicronucleate Cells

In ciliates, the MIC represents the germline nucleus and is genetically silent during vegetative growth. However, it has been shown in various species that MIC removal results in an immediate decrease in the rate of cell reproduction or can cause cell death [58,59,60]. These observations suggested that the MIC is essential for optimal vegetative growth, possibly by expressing a few MIC-specific key genes that are absent from the MAC [61,62]. This hypothesis is, however, contradicted by the fact that amic ciliates collected from the wild grow normally in laboratory cultures. Amic cells have been found in *Tetrahymena* [62], *Stylonychia* [63], *Oxytricha* [64], and, rarely, in *Euplotes* [65,66].

In the present study, we discovered amic cells in a wild-type strain and in offspring clones of *E. raikovi*. The *E. raikovi* amic cells have the same growth rate and viability as the mic cells. In addition, amic cells are able to form mating pairs efficiently, regardless of whether the other partner is micronucleate or not. During conjugation between one mic and one amic cell, the migratory pronucleus of the mic cell does not migrate to the amic cell but fuses with the stationary one to generate the synkaryon in a process similar to autogamy [67], and then undergoes a regular developmental process. In the amic mating cell, the parental MAC splits into several fragments of polymorphic bodies, which then fuse to regenerate a complete macronucleus. Conjugation also occurs between two amic cells: although no nuclear changes were observed during the process, cells remain united for 3–4 h before separation.

Conjugation between mic and amic cells was also reported in *Tetrahymena* [68]. In *Tetrahymena*, the gametic pronucleus from the normal mic cell enters into the amic partner, resulting in an identical 100% homozygous micronucleus in both cells. Their original macronucleus is retained until next round of conjugation, which will produce completely homozygous progeny. Therefore, amic cells in *Tetrahymena* are a valuable genetic tool that is used for various genetic applications [69]. This process differs in *E. raikovi*, where the migratory pronucleus very likely remains in the mic cell and fuses with the stationary one to form the synkaryon. However, we cannot exclude the possibility that the synkaryon is from the stationary pronucleus only while the migratory pronucleus is degraded. In any case, like in *Tetrahymena*, amic cells of *E. raikovi* may be used for some genetic applications, e.g., construction of homozygous strain from the mic cells through conjugating with the amic cells.

In conclusion, the nuclear events and the time needed for each step during conjugation in the marine species *Euplotes raikovi* were investigated in detail. Importantly, the posterior fragment of the parental MAC fuses into the new MAC, which provides proof that the residual parental DNA may have a direct influence on the new MAC, except for some indirect influence revealed in other ciliates. Moreover, we isolated amicronucleate cells of *E. raikovi* for the first time, which can mate with both micronucleate and amicronucleate cells. The amic cells will be valuable genetic tools that are used for various genetic applications. Further investigations at the molecular level are needed to better understand the roles of MIC and MAC genomes in the sexual process, the roles of parental MAC in the new MAC development, and how an amic cell is able to regenerate a functional and complete MAC genome from the fragments of the parental MAC.

## Figures and Tables

**Table 1:** Mating interactions between strains used in this study. For each cell combination, the intensity of mating interactions is indicated, from absent (−), 40% (++), to more than 70% (+++).

Strains	U	V	F1	I	XIII
**U**	−	++	+++	++	+++
**V**		−	−	++	−
**F1**			−	++	−
**I**				−	+++
**XIII**					−

## Supplementary Materials

The following are available online at https://www.mdpi.com/2076-2607/8/2/162/s1.
