# Supplementary material for: Conjugation in Euplotes raikovi (Protista, Ciliophora): New Insights into Nuclear Events and Macronuclear Development from Micronucleate and Amicronucleate Cells
Source: Microorganisms. 2020 Jan 23;8(2):162. doi: 10.3390/microorganisms8020162 (PMC7074782; doi:10.3390/microorganisms8020162)
Supplement: Supplementary file 1 [file microorganisms-08-00162-s001.pdf]

Supplementary figure

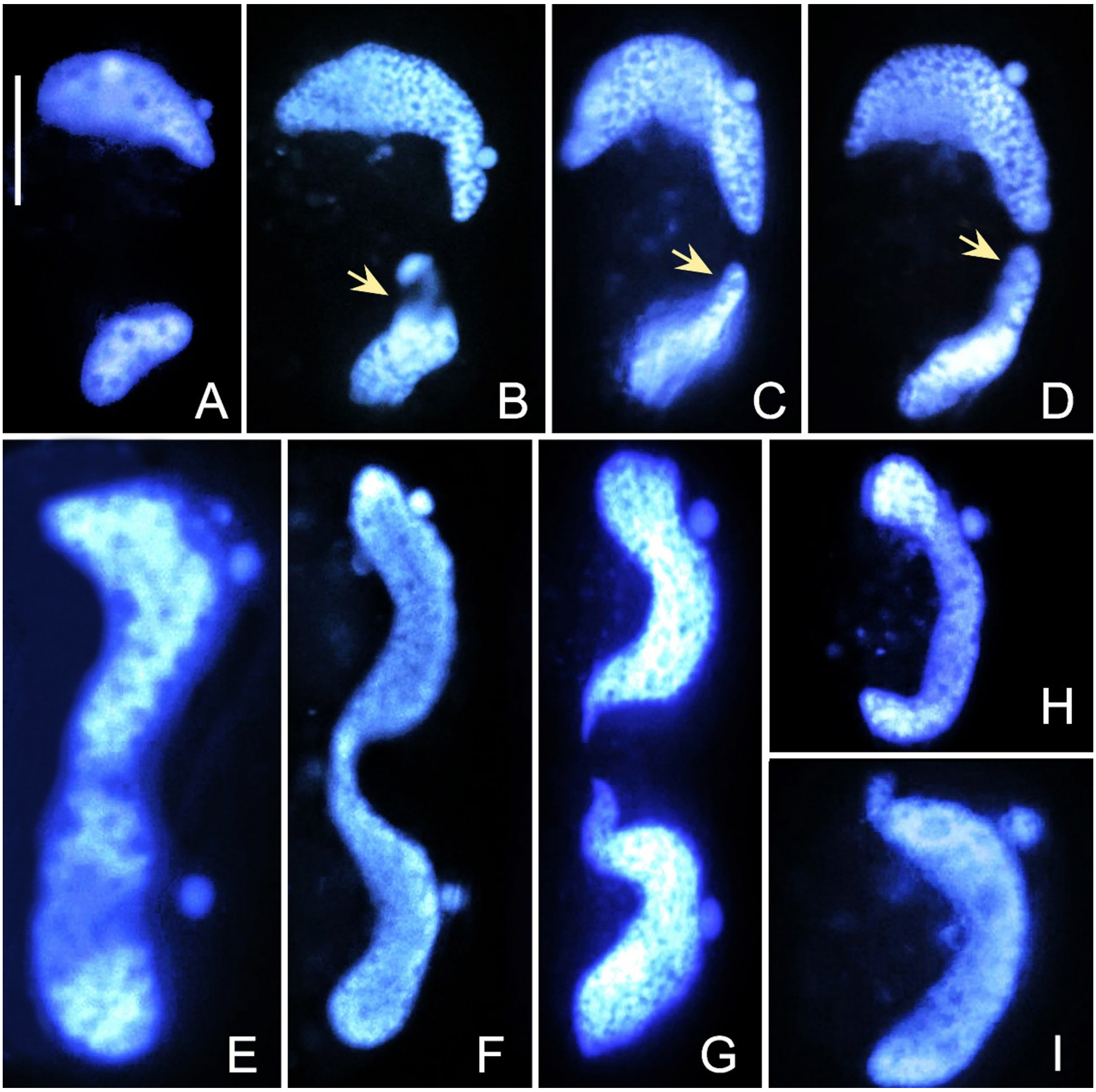

Figure S1. Nuclear events during new MAC development and the first postconjugational cell division in *Euplotes raikovi*. (A-D) Both the parental MAC (indicated in yellow arrows) and the MAC anlage increase in size, fuse together, and finally develop into the new MAC. (E-I) Nuclear events during the first postconjugational cell division. There is no observations of the degeneration of the fused parental MAC. Scale bar = 20 μm.
